# Nanotomographic evaluation of precipitate structure evolution in a Mg–Zn–Zr alloy during plastic deformation

**DOI:** 10.1038/s41598-020-72964-x

**Published:** 2020-09-30

**Authors:** Berit Zeller-Plumhoff, Anna-Lena Robisch, Daniele Pelliccia, Elena Longo, Hanna Slominska, Alexander Hermann, Martin Krenkel, Malte Storm, Yuri Estrin, Regine Willumeit-Römer, Tim Salditt, Dmytro Orlov

**Affiliations:** 1 Institute for Materials Research, Division of Metallic Biomaterials, Helmholtz Zentrum Geesthacht, Max-Planck-Straße 1, 21502 Geesthacht, Germany; 2 Institut für Röntgenphysik, Georg-August-Universität Göttingen, Friedrich-Hund-Platz 1, 37077 Göttingen, Germany; 3 Instruments & Data Tools Pty Ltd, PO Box 2114, Rowville, VIC 3178 Australia; 4 Institute for Materials Research, Division of Materials Physics, Helmholtz Zentrum Geesthacht, Max-Planck-Straße 1, 21502 Geesthacht, Germany; 5 Institute for Materials Research, Division of Materials Mechanics, Helmholtz Zentrum Geesthacht, Max-Planck-Straße 1, 21502 Geesthacht, Germany; 6 Diamond Light Source Ltd., Diamond House, Harwell Science and Innovation Campus, Didcot, OX11 0DE UK; 7 Department of Materials Science and Engineering, Monash University, Clayton, VIC 3800 Australia; 8 Department of Mechanical Engineering, The University of Western Australia, Crawley, 6009 Australia; 9 Division of Materials Engineering, Department of Mechanical Engineering, LTH, Lund University, P.O. Box 118, 22100 Lund, Sweden

**Keywords:** Materials science, Biomaterials, Techniques and instrumentation, Imaging techniques

## Abstract

Magnesium and its alloys attract increasingly wide attention in various fields, ranging from transport to medical solutions, due to their outstanding structural and degradation properties. These properties can be tailored through alloying and thermo-mechanical processing, which is often complex and multi-step, thus requiring in-depth analysis. In this work, we demonstrate the capability of synchrotron-based nanotomographic X-ray imaging methods, namely holotomography and transmission X-ray microscopy, for the quantitative 3D analysis of the evolution of intermetallic precipitate (particle) morphology and distribution in magnesium alloy Mg–5.78Zn–0.44Zr subjected to a complex multi-step processing. A rich history of variation of the intermetallic particle structure in the processed alloy provided a testbed for challenging the analytical capabilities of the imaging modalities studied. The main features of the evolving precipitate structure revealed earlier by traditional light and electron microscopy methods were confirmed by the 3D techniques of synchrotron-based X-ray imaging. We further demonstrated that synchrotron-based X-ray imaging enabled uncovering finer details of the variation of particle morphology and number density at various stages of processing—above and beyond the information provided by visible light and electron microscopy.

## Introduction

Magnesium (Mg) is one of the most potent metals for the modern sustainable living due to its abundance in the Earth’s crust, attractive property profile and recyclability perspective. The low density of Mg makes it appealing as a structural material, especially for light-weight mobility solutions. In the automotive sector, the historically longest utilization of Mg alloys in cast grades^1^ has been expanding recently to include precipitation-hardenable^2^ wrought alloys^3^. An overview of the most recent applications of automotive parts from Mg alloys in serial production including a Virtual Demonstrator can be found in reference^4^. A further potential application of Mg-based alloys is in medical devices. The relatively low toxicity of biodegradation products of Mg alloys makes them to promising candidate materials for bioresorbable medical implants and vascular stents^5^. The structural properties of Mg can be very efficiently controlled through alloying and thermo-mechanical treatment aimed at solid-solution, precipitate and strain hardening^2^. The ability to tailor the degradation rate remains pivotal to facilitate widespread applicability of Mg and its alloys in medical applications, as an exceedingly fast degradation can lead to unacceptable results, such as impairment of cell adhesion by ensuing hydrogen evolution, thus hindering bone growth and implant stability^6,7^, as well as increased cell apoptosis^8^.

As the kinetics of degradation is not only a function of the surface properties of the alloy but also depends on its bulk microstructure, the characterisation of the latter is of critical significance. This is especially the case when a radical variation of microstructure occurs during complex metal processing employed to improve the mechanical characteristics of Mg alloys. In addition to changes in the grain size and texture, severe plastic deformation associated with such processing may also bring about significant transformations in the population of intermetallic particles^8^. Traditional techniques of microstructure characterisation, such as scanning and transmission electron microscopy, provide insights into the local microstructure and, being surface-sensitive or limited to specimen thicknesses of the order of 100 nm, respectively, they may be insufficient to reveal fine detail of the intermetallic particle evolution over larger spatial scales. A potent complementary approach is based on synchrotron-based tomographic X-ray imaging techniques for three-dimensional analysis with high spatial and temporal resolution, which may also serve as an alternative for two-dimensional imaging techniques of similar resolution. While the resolution of X-ray imaging cannot reach the level achieved by TEMs, these techniques allow investigating much larger sample volumes, which yields a different but nonetheless critical information about the sample structure.

This paper reports the results of our first efforts in adopting nanotomographic X-ray imaging for the analysis of precipitate structure evolution in Mg alloys. Specifically, we use holotomography and transmission X-ray microscopy (TXM). Holotomography has previously been used to study the microstructure of various alloys^9,10^ as well as the evolution of phases during heat treatment^11^ and selective laser melting^12^, including its correlation to stress corrosion cracking^13^. Holotomography and TXM are full-field imaging techniques that do not necessitate lateral scanning of the sample and are therefore particularly well suited for tomography as well as time resolved studies.

As a representative material for the present study, we select a commercial wrought Mg–Zn–Zr alloy ZK60 in three microstructural states displaying significant differences in precipitate structure. The material was processed by a single-pass integrated direct extrusion (DE) and parallel-channel equal-channel angular pressing (PC-ECAP). This processing resulted in significant improvement of the key performance characteristics of the material^14–16^, which was primarily associated with a dramatic evolution of its precipitate structure^17,18^. This evolution was previously studied extensively by means of scanning and transmission electron microscopy (SEM and TEM, respectively) and small-angle X-ray scattering (SAXS) ^17,18^. It was found that agglomerated colonies of primary Mg–Zn and Zn–Zr intermetallic particles (e.g. MgZn_2_, Mg_4_Zn_7_ and Zr_2_Zn_3_) at grain boundaries and in proximity to triple junctions in the initial material condition break up, redistribute and most likely re-solutionise at the intermediate stage of processing, i.e. during the direct extrusion step^18^. Secondary precipitates such as basal platelets and c-axis rods also appeared significantly finer than those typically observed in alloys of the Mg–Zn system after static ageing^17^. The precipitate structure observed after the final processing stage, viz. severe plastic deformation by PC-ECAP, exhibited further reduction in the length scale of features and a general homogenization of the microstructure and chemical composition as well as the formation of nano-scale prismatic platelets in a statistically significant amount. Zn–Zr intermetallics occurred primarily along plastic flux lines throughout the microstructure. These reports have established an excellent basis for the correlative analysis of the potential of X-ray imaging in the study of precipitate structure evolution in Mg alloys.

Building on the work described above, this study demonstrates the usefulness of 3D X-ray imaging of Mg alloys for investigating precipitate morphology quantitatively. It highlights advantages of these imaging techniques in terms of statistical significance while recognizing limitations in resolution as compared to electron microscopy techniques.

## Materials and methods

### Materials processing and specimen preparation

Commercial magnesium alloy ZK60 having a chemical composition of Mg–5.78Zn–0.44Zr (in wt.%) was investigated. The material was processed by a single-pass PC-ECAP, as briefly described above and reported in detail elsewhere^14–16^. Various precipitate states were sampled at each stage of processing, i.e. initial (before extrusion), intermediate (after DE, i.e. past the extrusion die section of the rig) and final (after the entire processing cycle encompassing DE and PC-ECAP).

Needle-shaped specimens of 100–160 µm diameter were produced from each material sample by cutting rods with the cross-sectional dimensions of 500 µm × 500 µm using a diamond-wire saw and oil lubricant followed by electropolishing to the desired cross-section size. The long axis of the needles was perpendicular to the extrusion direction (ED) for specimens at the intermediate stage and parallel to ED at the final one. The maximum needle diameter was chosen according to the attenuation length of Mg at an X-ray energy of E = 8 keV, *i.e.,* the depth at which the transmitted beam intensity drops to 1/e of the incoming beam.

### Imaging

#### Holotomography

Needles prepared from material at each processing stage were first imaged at the Göttingen Instrument for Nano-Imaging with X-rays (GINIX) at beamline P10 at Deutsches Elektronen Synchrotron (DESY). The instrument has been designed for near-field phase contrast imaging (also known as in-line holographic imaging) in a high magnification / high resolution setting^19^. In the present experiment, a lithographically defined silicon waveguide, as described in reference^19^, was used to achieve an effective source with size below 20 nm. The holotomography method requires several tomographic scans of the same sample to be performed at different propagation distances. Holograms taken at different propagation distances are used for phase retrieval, resulting in two-dimensional (2D) images of the projected phase shift of the sample. Finally, the projected phase shift of each angular position is used for tomographic reconstruction of the three-dimensional (3D) image of the sample at high resolution^20^.

An X-ray energy of E = 8 keV was used for image acquisition with a spacing of focal spot and detector of 5.178 m. In holotomography, the magnification and hence the field of view and resulting voxel size can be varied by choosing appropriate source-to-sample distances due to the cone beam geometry of the setup. A scintillator-based fibre-coupled sCMOS detector (Photonic Science, UK) with 6.5 µm pixel size and Gadox scintillator was used. Firstly, an overview scan of the needle was performed using propagation distances between focal spot and sample of 150, 155, 170 and 190 mm and resulting in a reconstructed voxel size of 188.3 nm. Secondly, a region of interest (ROI) scan was performed at propagation distances of 40, 41, 45 and 50 mm with a final voxel size of 50.21 nm. The phase retrieval and tomographic 3D image reconstruction was performed using the holotomography toolbox^21^ in Matlab R2018b (The MathWorks Inc., USA). For each sample, the optimal phase retrieval algorithm was determined based on visual inspection. These were either based on the contrast transfer function (CTF)-formalism^20,22^ or on an implementation of the non-linear Tikhonov approximation^21,23^. Table 1 summarizes the selected algorithm and the ratio between phase shifting (δ) and absorbing (β) components of the index of refraction, i.e. δ/β-ratio required for the CTF-formalism. The tomographic reconstruction was performed using filtered back projection.

**Table 1:** Summary of selected phase-retrieval algorithms and δ/β-ratios for each holotomography scan.

	Full-width scan			ROI scan		
	Initial	Intermediate	Processed	Initial	Intermediate	Processed
Phase-retrieval algorithm	Non-lin. Tikhonov	CTF	CTF	CTF	CTF	CTF
δ/β-ratio	–	0.2	0.2	0.25	0.25	0.25

#### Transmission X-ray microscopy (TXM)

To gain further statistical information on particle evolution between initial and intermediate processing (past DE) stages, three selected needle specimens for each condition were imaged using the transmission X-ray microscopy setup at the Diamond Manchester Imaging Branchline I13-2 at Diamond Light Source^24^. An energy of E = 12 keV was selected using a double multilayer monochromator (Mo/B_4_C coating, ΔE/E = 0.27%). The sample was illuminated using a beam shaping condenser with square fields^25^. A Fresnel zone plate (FZP) with an outermost zone width dr = 50 nm was used as objective and Zernike phase rings were installed in the back-focal plane of the FZP to enable phase contrast imaging. All optics were designed and fabricated in the X-ray Optics and Applications group of Paul-Scherrer-Institut (Switzerland).

A Hamamatsu C12849-101U camera with a sCMOS chip with 6.5 µm pixel size and a 10 µm Gadox scintillation layer was used as detector system. The measured X-ray magnification factor was M = 187.3, yielding an effective pixel/voxel size of 34.7 nm.

A region of interest scan of each needle was performed at a random location within each needle. Reconstructions were performed in the open-source Savu framework^26^ using the Tomopy reconstruction package^27^.

#### Image processing and data analysis

The reconstructed images were binned twice in all directions using Fiji/ImageJ^28^ to reduce image noise. Furthermore, images obtained using TXM were previously filtered using an iterative non-local means filter^29^. The trainable WEKA plugin^30^ in Fiji was used for a first segmentation of the datasets, which was refined manually in Avizo 9.4.0 (FEI SAS, Thermo Scientific, France). The 3D Objects Counter in Fiji was then employed to analyse the connected particles identified in the segmentation in terms of their surface area $S$ and volume $V$. The minimum size of a particle to be taken into consideration was 8 (2 × 2 × 2) voxels, which corresponds to a volume of 2.67·10^–3^ µm^3^ at the smallest voxel size. The distance between particles was computed as the Euclidean distance between their centroids, which were given by the 3D Objects Counter. Additionally, the particle analyser of the BoneJ plugin^31^ was used to compute the Feret diameter of each particle in the ROI scans, which relates to the largest distance between two point in the particle. Finally, Matlab 2018a was used to compute the morphological parameters and distributions, and to create the figures presented in this article. Normal distributions were fitted to the distance distributions of particles to determine the mean value and standard deviation of the interparticle distances.

Sphericity was computed for each particle as ${C}_{p}=\frac{6{\pi }^{0.5}V}{{S}^{1.5}}$; if ${C}_{p}=1$ the particle is considered spheroidal, whereas ${C}_{p}=0$ represents a plate-like shape^32^. Surface area $S$ and volume $V$ were determined as stated above.

For the full-width scans, the sample outline was segmented in Avizo using a region growing algorithm, with automated closing of remaining holes. To avoid the influence of strong image artefacts at the needle edges, the segmented outline was eroded using a cubic window of a size of 20 pixels. Similarly, to avoid the influence of artefacts from the top edge, the top 100 slices of the specimens were excluded from the analysis.

## Results and discussion

### Phase retrieval and 3D volume reconstruction

**Figure 1:**
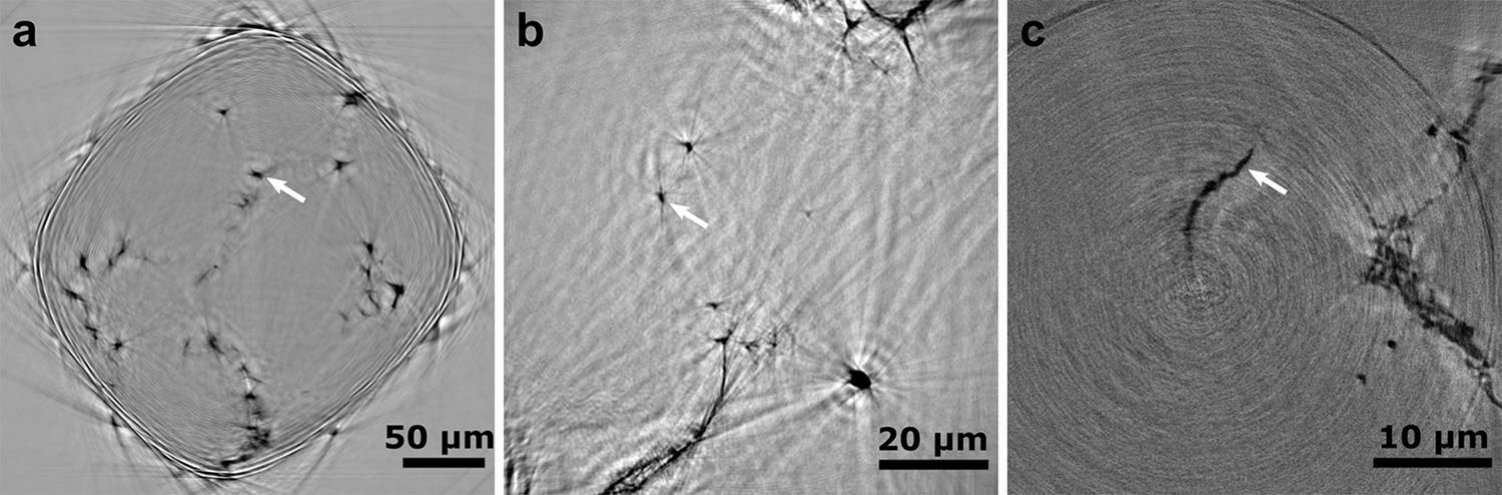
Cross-section slices from the tomographic reconstructions of samples in the initial state, prior to processing, after a full-width (**a**) and a ROI (**b**) scans performed with GINIX at P10, DESY; scale bars are 50 µm and 20 µm, respectively; (**c**) shows a slice of a ROI scan imaged at I13-2, scale bar is 10 µm. White arrows indicate exemplary particles that are visible in the image.

Figure 1 shows slices from 3D specimen reconstructions at the initial processing stage for the full-width (a) and ROI scans (b) performed at GINIX with 8 keV and a ROI scan (c) performed with TXM at I13-2 using 12 keV. The precipitates are visible in black within the lighter Mg matrix, as indicated by exemplary white arrows. Slices from the holotomography scans in Figs. 1a,b have higher contrast ranges than those from TXM. At the same time, all the images contain phase and 3D reconstruction artefacts that are (i) fringe-like at the specimen-air interface in Fig. 1a, streak-like around standalone precipitates in Fig. 1b, and ring-type in Fig. 1c. The streak artefacts in holotomography images are due to absorption, which is more prominent for low energies. Furthermore, the fringes arise from the fact that strong phase gradients are not compatible with the assumptions made for linearized phase retrieval. Future improvements including iterative phase retrieval methods as well as experiment optimizations such as adjustment of photon energy, embedding the sample in a homogeneous matrix or using a needle diameter larger than the illumination field may alleviate this issue.

The scale and morphology of retrieved particles, and their comparison with prior studies, also suggest that only primary incoherent particles similar and consistent with those identified with SEM in prior studies^17,18^ are visible. The current resolution of X-ray imaging still does not allow the visualisation of coherent precipitates found in this material with TEM and SAXS earlier^17,18^.

### Visual inspection and qualitative analysis of reconstructed particles

**Figure 2:**
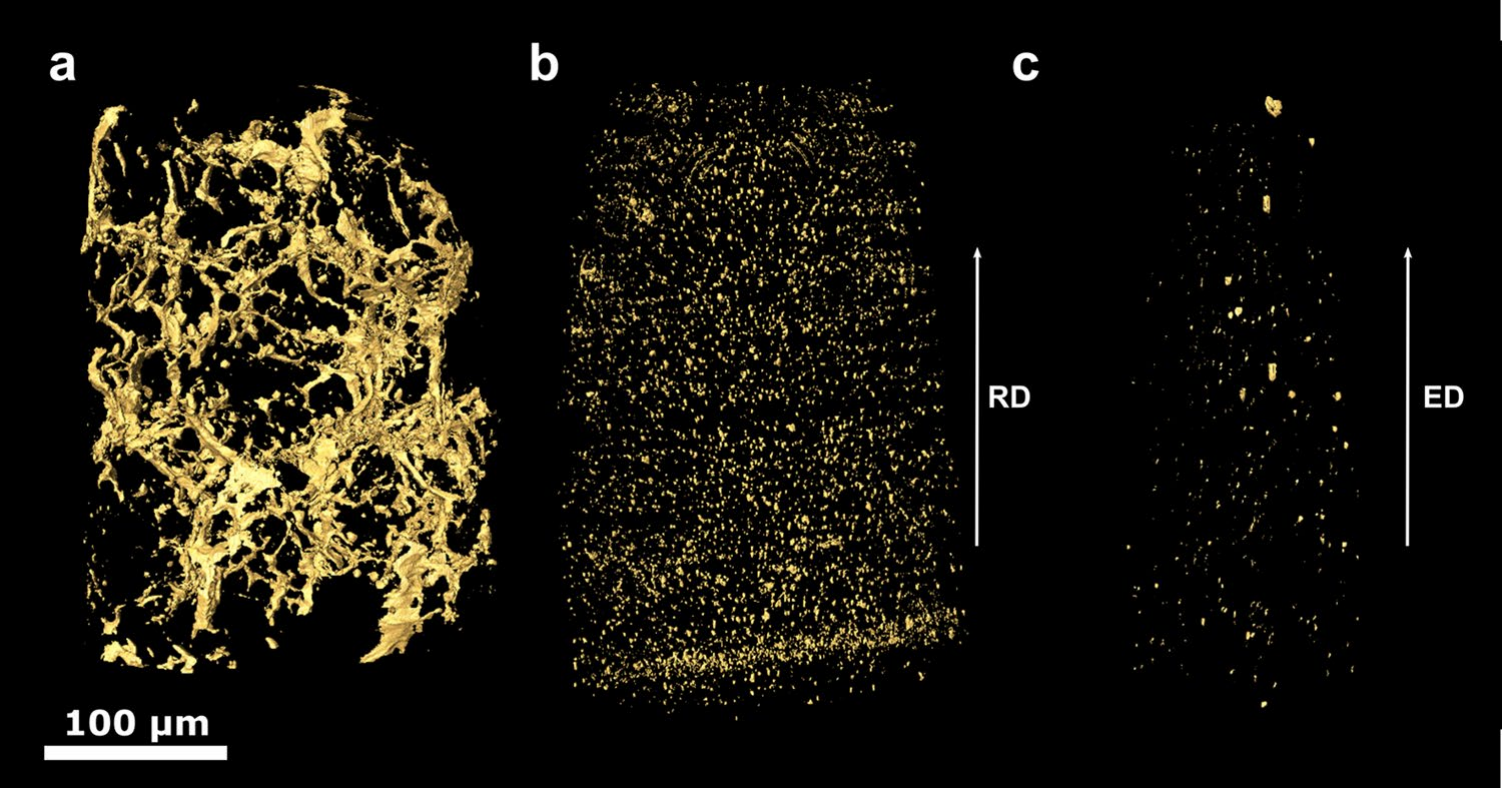
3D renderings of segmented particles in the full-width scans of samples at the initial (**a**), intermediate (**b**) and final (**c**) processing stages imaged with GINIX at P10, DESY. The scale bar is 100 µm. Arrows labelled ED and RD show the extrusion and radial specimen directions, respectively.

**Figure 3:**
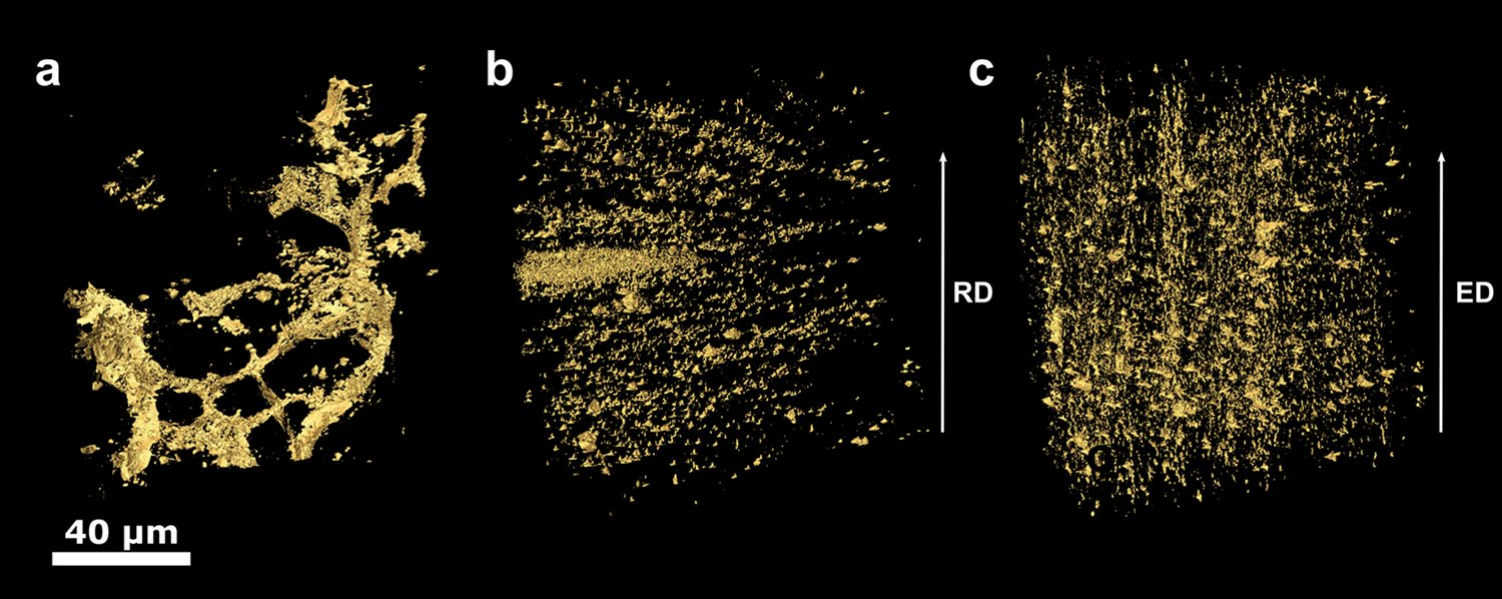
3D renderings of segmented particles in the ROI scans of samples at the initial (**a**), intermediate (**b**) and final (**c**) processing stages imaged with GINIX at P10, DESY. The scale bar is 40 µm. Arrows labelled ED and RD show the extrusion and radial specimen directions, respectively.

Figures 2 and 3 display the volume rendering of segmented particles in full-width and ROI specimen scans, respectively, at the initial (a), intermediate (b) and final (c) processing stages imaged with GINIX at P10, DESY. At the initial stage, Figs. 2a and 3a, a sparse connected network of agglomerated primary Mg–Zn particles is visible. Size and morphology of large openings in this network is consistent with matrix grains identified earlier^20^. The intermediate stage, Figs. 2b and 3b, shows dispersed particles with a more spherical morphology. While a rather homogeneous distribution of particles in a volume corresponding to several initial grains is seen, some oblong areas with a denser preferential distribution of particles along the extrusion direction can also be found. It is worth mentioning that qualitatively the precipitate structures of full-width scans in Fig. 2a,b and respective ROI scans in Fig. 3a,b are very similar, the only difference being finer details discernible in the latter.

A distinctly different picture is seen for the fully processed material after combined DE and PC-ECAP deformation. In the full-width scan, Fig. 2c, particles appear very different from those in ROI scan, Fig. 3c. The former reveals a very low number density of particles in a wide size range distributed homogeneously within the scan volume. Larger particles in Fig. 2c tend to be slightly elongated in extrusion direction. By contrast, the ROI scan in Fig. 3c shows the highest number density of particles. Most of them are rather homogeneously distributed while some are still agglomerated in irregular-shape areas up to a few micrometres in size or elongated in ED.

ROI scans of samples at the initial and intermediate processing stages imaged with TXM at I13-2 are presented in the supplementary figures. All observations are consistent between each other and with earlier 2D SEM examinations on representative sample Sections^23^.

### Quantitative analysis of precipitate structure evolution in 3D

#### Particle volume distribution

**Figure 4:**
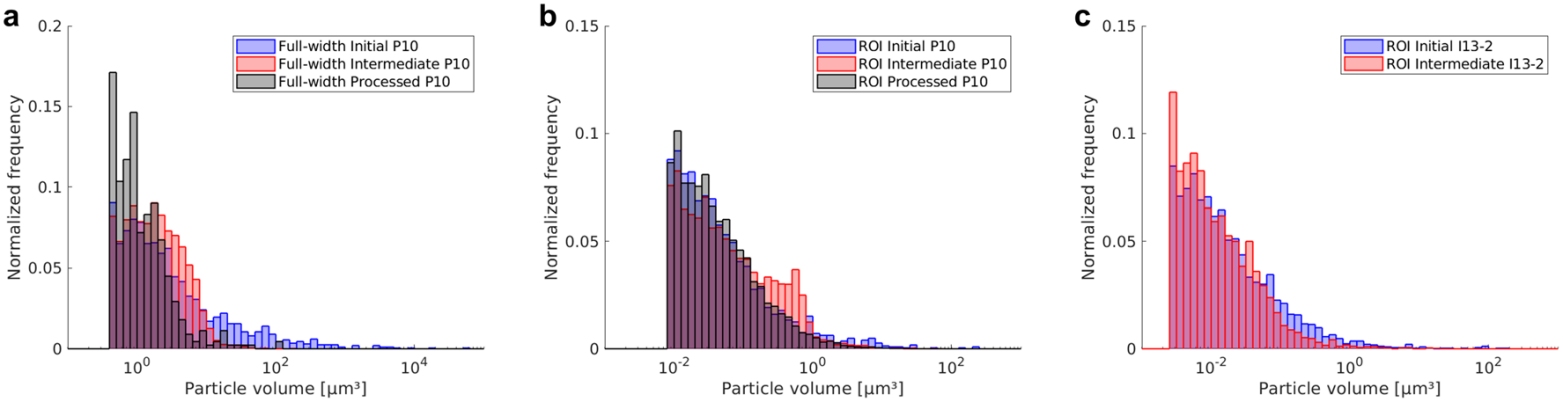
Distribution of particle volumes for full-width (**a**) and ROI (**b**) scans from GINIX at P10, DESY and ROI scans from I13-2, Diamond (**c**). Blue corresponds to the initial state, red to the intermediate stage and grey to the final processing stage.

Figure 4 shows the distribution of particle volumes for the full-width (a) and ROI (b) scans from GINIX at P10, DESY and ROI scans from I13-2, Diamond (c). Processing to the intermediate stage leads to a decrease in volume and an increase of particle number densities (frequencies). A further shift to smaller particle volumes is seen from the intermediate to the processed stage. The trends are consistent among all three scan types while specific frequencies of volume fractions vary modestly. The volumes of ROI scans generally reach lower values due to a decrease in voxel size.

#### Particle sphericity versus volume

**Figure 5:**
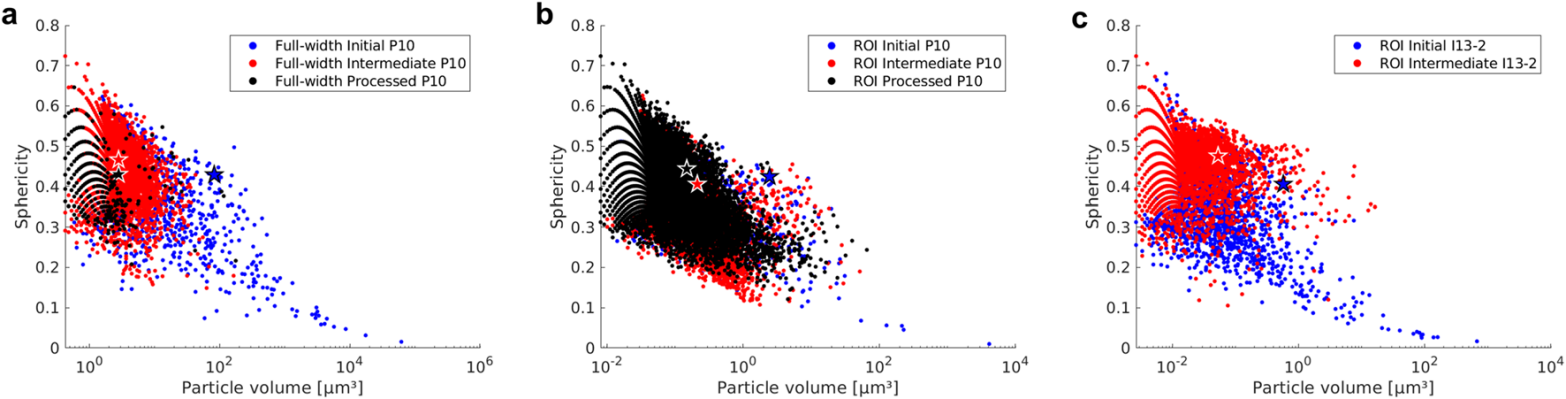
Particle sphericity versus*.* volume mapping of full-width (**a**) and ROI (**b**) scans from GINIX at P10, DESY and ROI scans from I13-2, Diamond (**c**). Blue corresponds the initial state, red to the intermediate stage and black to the final processing stage. Stars indicate the respective data centroids.

Figure 5 shows the particle sphericity versus volume for full-width (a) and ROI (b) scans from GINIX at P10, DESY, and ROI scans from I13-2, Diamond (c). Stars in the figure indicate the data centroids. The largest particles visible at the initial processing stage display a plate-like morphology, i.e. low sphericity values, as can also be observed qualitatively in the 3D renderings in Figs. 2 and 3. Deformation processing leads to a shift towards more spherical objects at similar particle volumes (upwards shift of the point cloud). The shift in sphericity from the initial to the intermediate processing stage is strongest in the I13-2 ROI scans, Fig. 5c, which may be attributed to the absence of streak artefacts in the image, see Fig. 1c. The difference between intermediate and final processing stages in Fig. 5a,b is small but the trend towards further refining and spheroidization of particles with strain increase is still evident. However, the point cloud for the full-width scan of the processed stage is very sparse, due to a shift of particle sizes below the resolution of this scan. The periodicity in sphericity at low particle volumes visible in all three figures is due to the fewer distinct shapes that particles of lower volume may take. There are only few ways in which the voxels belonging to a particle of a certain low volume can be arranged. This results in distinct surfaces and therefore sphericity values.

#### Particle Feret diameter versus volume (fraction)

**Figure 6:**
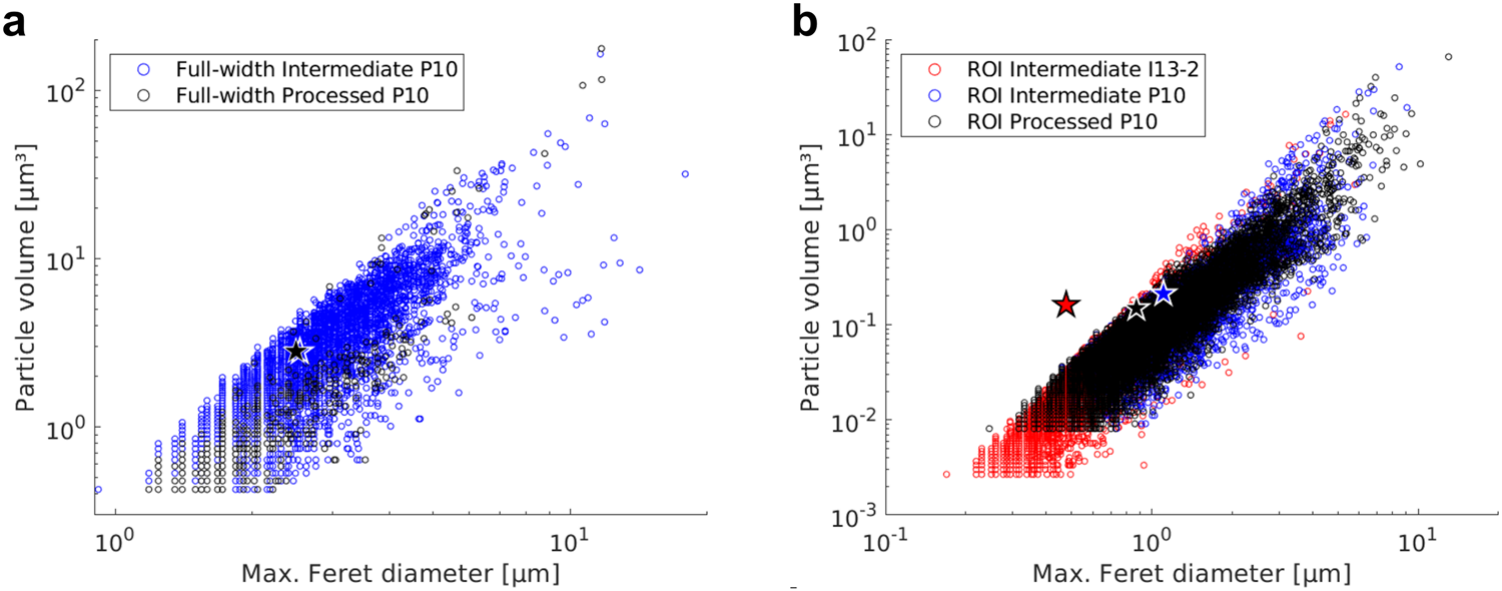
Maximum Feret diameter versus particle volume for the full-width (**a**) and the ROI (**b**) scans. Blue and black show the intermediate and the processed stages imaged with GINIX at P10, while red shows the intermediate stage imaged at I13-2. Stars indicate the respective data centroids.

Figure 6 displays the Feret diameter of the particles in the full-width (a) and the ROI (b) scans versus the particle volume. Data for the initial material condition were excluded from this figure, as the particles are very large and irregularly shaped. While the range of maximum Feret diameters of particles is rather large for both material conditions, the cloud at the final processing stage appears to have a lower spread. Large stars in the figure indicate the centroids of maximum particle diameter variations. They show a clear shift towards smaller particle dimensions with the increase of strain level from the intermediate to the final processing stage in the ROI scans. This shift is less strong in the full-width scans due to their lower resolution. The difference between particle dimension centroids in the intermediate-stage data from GINIX (P10) and I13-2 ROI scans, Fig. 6b, can be explained by a slightly lower number of artefacts along with an increase in image resolution in the I13-2 ROI, and therefore the visualisation of smaller particles.

#### Interparticle distance

**Figure 7:**
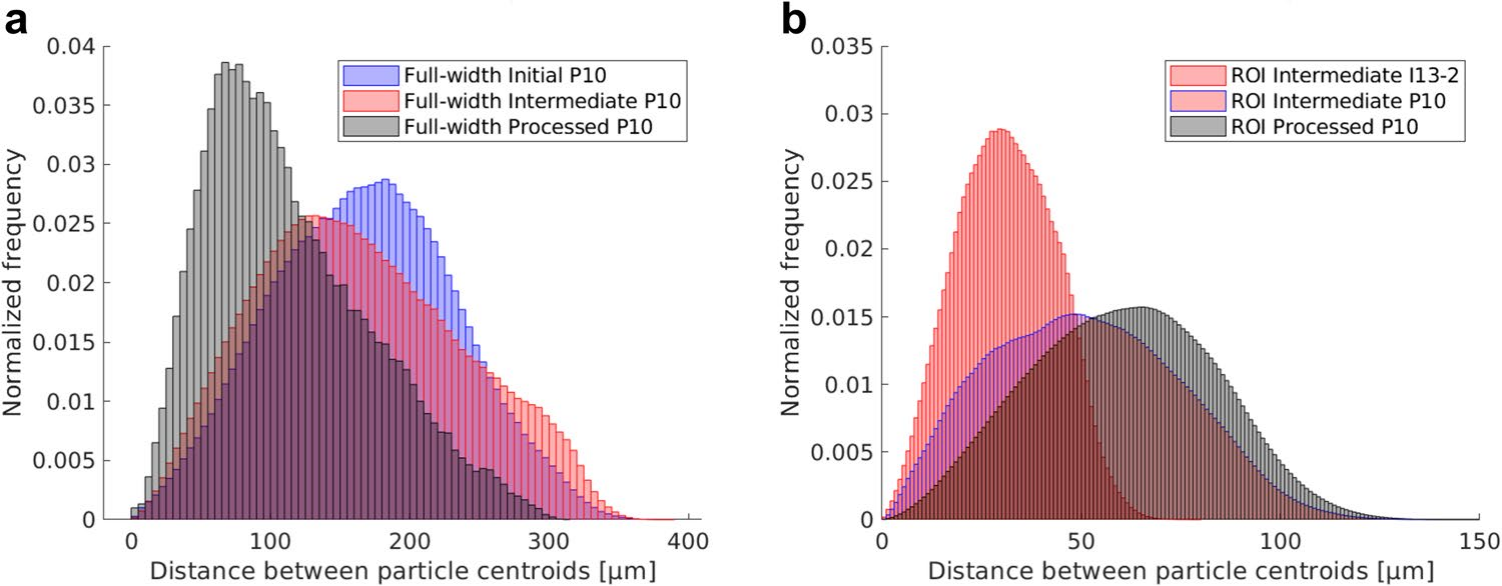
Interparticle distance distribution for the full-width (**a**) and the ROI (**b**) scans. Blue, red (with blue edges) and grey represent the initial, intermediate and the processed stages imaged with GINIX at P10, while red corresponds to the intermediate stage imaged at I13-2.

The interparticle distance distributions obtained from the Euclidean distances between the particle centroids are displayed in Fig. 7 for the full-width (a) and the ROI (b) scans. Again, the initial condition from ROI scans is omitted. In the initial condition of full-width scans, the interparticle distances have a normal distribution with a mean value of 164.82 ± 70.72 µm, see blue bars in Fig. 7a. Deformation processing to the intermediate stage leads to the splitting of the distribution with the original peak slightly shifting toward shorter distances (127.19 ± 57.65 µm) because of the emergence of a new peak at ~ 300 µm, see red bars in Fig. 7a. Following further processing decreases the mean particle distance further (68.89 ± 30.08 µm). A different shift of mean interparticle distances towards larger values upon the increase of strain from the intermediate (49.16 ± 26.79 µm) to the final processing stage (60.45 ± 25.04 µm), can be found from the comparison of violet and grey bar distributions in the data from ROI scans in Fig. 7b. This difference may however also be due to the difference in considered sample volume—while the sample volume of P10 ROI scans was constant at 8.15·10^–4^ mm^3^ (1.29·10^–4^ mm^3^ for I13-2 ROI scans), sample volumes for full-width scans varied from 1.27·10^–3^ to 0.99·10^–3^ and 0.3·10^–3^ mm^3^ for initial, intermediate and final processing stage, respectively. The mean particle distance in the ROI scan at I13-2 (30.95 ± 13.75 µm) is lower than that at P10 since smaller particles are detectable in the former case, as discussed earlier, and due to the generally smaller field of view.

When the data presented in Fig. 7b is considered along with that in Fig. 4, it can be concluded that the reduction of the particle size correlates well with the increase of interparticle spacing. A bias in the quantitative statistical analysis may be introduced if the ROI happens to include localised denser particle fluxes during deformation, see for instance Figs. 2b and 3b. Therefore, a qualitative examination of 3D reconstructions needs to be conducted prior to statistical analysis to eliminate a possible bias. Another factor affecting the interparticle distance distribution can be associated with a shift of particle sizes below the resolution limit of a specific technique or particle dissolution in the matrix. In such cases, correlative imaging with higher-resolution techniques, e.g. TEM, must be carried out.

#### Benefits of X-ray imaging for the analysis of precipitate structure in Mg alloys

The results of this work provide further confirmation of the earlier findings based on optical and electron microscopy. Capitalizing on an already comprehensively studied material, both X-ray imaging techniques used in this study, *i.e*. holotomography and TXM, provide more statistically significant qualitative and quantitative 3D information about the evolution of the precipitate structure in modern Mg alloys. Moreover, the synchrotron-based X-ray imaging is significantly less demanding with regard to specimen preparation compared to the techniques used previously. The present study also lays a foundation and opens opportunities for in situ studies of precipitate structure evolution in Mg alloys. Thereby, structural changes can be linked to the temporal scale of applied forces, elevated temperature, etc. and enable the derivation of mechanisms corresponding microstructure evolution.

Compared to far-field coherent diffractive imaging and ptychography^33^, the detector signal in holotomography is much more spatially uniform. A comparison of our results gained from holotomography and TXM imaging suggests that for Mg alloys differences in the capabilities of the two techniques are minor. The spatial resolution of ROI scans in both techniques seems to be at the same length scale of about 100 nm. This is still two orders of magnitude coarser than TEM, thus limiting the analysis to primary precipitate particles only. Nevertheless, the pace of development in the available X-ray imaging techniques along with the introduction of new sources, such as MAX IV Lab synchrotron in Lund, promise a fast near-future improvement of the resolution to include the secondary coherent precipitates in Mg alloys too. At the same time, the detection limit by volume fraction in the 10^–7^ range is significantly better than that in powder diffraction and scattering based techniques typically providing ~ 0.1% (or 10^–3^) in the case of synchrotron radiation.

A noticeable advantage of holotomography over TXM is the capability to vary field of view and resolution via propagation distances without the need to realign optics as well as the quantitative phase contrast^34^, which enables to extract the electron density of the precipitates. The zoom capability is of high interest for in situ experiments including the selection of the appropriate experimental methodology for understanding the degradation mechanisms of precipitation-hardenable Mg alloys in aqueous media. It has been shown previously that synchrotron-based X-ray imaging enables *in situ* testing in aqueous media at sufficiently high temporal resolution^35,36^, yet the step towards in situ imaging of degradation of Mg alloys at the sub-micron scale still remains to be taken.

## Conclusions

The results presented have highlighted the benefits of holotomography and transmission X-ray microscopy. We believe to have provided a convincing demonstration that these state-of-the-art techniques help to close the gap between local high-resolution imaging methods, such as SEM and TEM, and the larger volume nanotomographic X-ray imaging. The particular case of Mg alloy with a rich deformation processing history, which resulted in complex precipitate structure evolution, offered a testbed for evaluating the potential of nanotomographic 3D X-ray imaging methods. Both three-dimensional imaging techniques trialed enable non-destructive testing along with quantitative statistical analysis of precipitate morphology and distribution with a spatial resolution better than 100 nm. These capacities will be further exploited for the testing of in situ degradation of magnesium alloys. We expect that further tuning of imaging parameters, notably the photon energy, will result in significant improvements of the image quality.

## Supplementary information

Supplementary legend

Supplementary video 1

Supplementary video 2

Supplementary video 3

Supplementary video 4

Supplementary video 5

Supplementary video 6

Supplementary figure

## References

[1] Mordike BL, Ebert T (2001). Magnesium: properties–applications–potential. Mater. Sci. Eng. A.

[2] Nie J-F (2012). Precipitation and hardening in magnesium alloys. Metall. Mater. Trans. A.

[3] Joost WJ, Krajewski PE (2016). Towards magnesium alloys for high-volume automotive applications. Scr. Mater..

[4] IMA. *Mg Applications: Automotive*. https://www.intlmag.org/page/app_automotive_ima (2020).

[5] Witte F (2008). Degradable biomaterials based on magnesium corrosion. Curr. Opin. Solid State Mater. Sci..

[6] Keim S, Brunner JG, Fabry B, Virtanen S (2011). Control of magnesium corrosion and biocompatibility with biomimetic coatings. J. Biomed. Mater. Res. B Appl. Biomater..

[7] Lorenz C (2009). Effect of surface pre-treatments on biocompatibility of magnesium. Acta Biomater..

[8] Dobatkin S (2019). Mechanical properties, biodegradation, and biocompatibility of ultrafine grained magnesium alloy WE43. Materials (Basel).

[9] Requena G (2009). Sub-micrometer synchrotron tomography of multiphase metals using Kirkpatrick-Baez optics. Scr. Mater..

[10] Landron C (2012). Non-destructive 3-D reconstruction of the martensitic phase in a dual-phase steel using synchrotron holotomography. Scr. Mater..

[11] Tolnai D, Requena G, Cloetens P, Lendvai J, Degischer HP (2012). Sub-micrometre holotomographic characterisation of the effects of solution heat treatment on an AlMg73Si3.5 alloy. Mater. Sci. Eng. A Struct. Mater..

[12] Barriobero-Vila P (2017). Inducing stable alpha + beta microstructures during selective laser melting of Ti-6Al-4V using intensified intrinsic heat treatments. Materials (Basel).

[13] Altenbach C (2020). Synchrotron-based holotomography and X-ray fluorescence study on the stress corrosion cracking behavior of the peak-aged 7075 aluminum alloy. J. Alloy. Compd..

[14] Orlov D, Raab G, Lamark TT, Popov M, Estrin Y (2011). Improvement of mechanical properties of magnesium alloy ZK60 by integrated extrusion and equal channel angular pressing. Acta Mater..

[15] Orlov D, Hockauf M, Meyer LW, Estrin Y (2013). Dynamic properties of an ultrafine-grained Mg–Zn–Zr alloy. Philos. Mag. Lett..

[16] Vinogradov A, Orlov D, Estrin Y (2012). Improvement of fatigue strength of a Mg–Zn–Zr alloy by integrated extrusion and equal-channel angular pressing. Scr. Mater..

[17] Orlov D (2014). Particle evolution in Mg-Zn-Zr alloy processed by integrated extrusion and equal channel angular pressing: evaluation by electron microscopy and synchrotron small-angle X-ray scattering. Acta Mater..

[18] Orlov D, Ralston KD, Birbilis N, Estrin Y (2011). Enhanced corrosion resistance of Mg alloy ZK60 after processing by integrated extrusion and equal channel angular pressing. Acta Mater..

[19] Bartels M, Krenkel M, Haber J, Wilke RN, Salditt T (2015). X-ray holographic imaging of hydrated biological cells in solution. Phys. Rev. Lett..

[20] Cloetens P (1999). Holotomography: Quantitative phase tomography with micrometer resolution using hard synchrotron radiation x rays. Appl. Phys. Lett..

[21] Lohse, L. M. *et al.* A phase-retrieval toolbox for X-ray holography and tomography. *J. Synchrotron Radiat.* **Accepted 19 February 2020**, In Press (2020). 10.1107/S1600577520002398 PMC7206550 32381790

[22] Zabler S, Cloetens P, Guigay JP, Baruchel J, Schlenker M (2005). Optimization of phase contrast imaging using hard x rays. Rev. Sci. Instrum..

[23] Davidoiu V, Sixou B, Langer M, Peyrin F (2011). Non-linear iterative phase retrieval based on Frechet derivative. Opt. Express.

[24] Storm, M., Döring, F., Marathe, S., David, C. & Rau, C. The Diamond I13 full-field transmission X-ray microscope: a Zernike phase-contrast setup for material sciences. *Powder Diffr.* doi:accepted (2020).

[25] Vartiainen I, Mokso R, Stampanoni M, David C (2014). Halo suppression in full-field x-ray Zernike phase contrast microscopy. Opt Lett.

[26] Wadeson, N. & Basham, M. Savu: A Python-based, MPI framework for simultaneous processing of multiple, N-dimensional, large tomography datasets. *arXiv*, https://arxiv.org/abs/1610.08015 (2016).

[27] Gursoy D, De Carlo F, Xiao X, Jacobsen C (2014). TomoPy: a framework for the analysis of synchrotron tomographic data. J. Synchrotron. Radiat..

[28] Schindelin J (2012). Fiji: an open-source platform for biological-image analysis. Nat. Methods.

[29] Bruns S, Stipp SLS, Sorensen HO (2017). Looking for the Signal: A guide to iterative noise and artefact removal in X-ray tomographic reconstructions of porous geomaterials. Adv. Water. Resour..

[30] Arganda-Carreras I (2017). Trainable Weka Segmentation: a machine learning tool for microscopy pixel classification. Bioinformatics.

[31] Doube M (2010). BoneJ: free and extensible bone image analysis in ImageJ. Bone.

[32] Ohser JM (2000). F.

[33] Miao J, Ishikawa T, Robinson IK, Murnane MM (2015). Beyond crystallography: DIffractive imaging using coherent x-ray light sources. Science.

[34] Salditt T (2015). Compound focusing mirror and X-ray waveguide optics for coherent imaging and nano-diffraction. J. Synchrotron. Radiat..

[35] Feyerabend, F. *et al.* in *SPIE Optical Engineering + Applications.* 99671X-99671X-99679 (International Society for Optics and Photonics).

[36] Zeller-Plumhoff B (2018). Quantitative characterization of degradation processes in situ by means of a bioreactor coupled flow chamber under physiological conditions using time-lapse SRµCT. Mater. Corros..

