# Supplementary material for: Nanotomographic evaluation of precipitate structure evolution in a Mg–Zn–Zr alloy during plastic deformation
Source: Sci Rep. 2020 Sep 30;10:16101. doi: 10.1038/s41598-020-72964-x (PMC7527343; doi:10.1038/s41598-020-72964-x)
Supplement: Supplementary file 1 — Supplementary legend [file 41598_2020_72964_MOESM1_ESM.docx]

**Supplementary Videos**

Videos of the 3D renderings from P10 scans are provided as supplementary material for better visualization of the 3D structure.
