# Supplementary material for: Nanotomographic evaluation of precipitate structure evolution in a Mg–Zn–Zr alloy during plastic deformation
Source: Sci Rep. 2020 Sep 30;10:16101. doi: 10.1038/s41598-020-72964-x (PMC7527343; doi:10.1038/s41598-020-72964-x)
Supplement: Supplementary file 8 — Supplementary figure [file 41598_2020_72964_MOESM8_ESM.docx]

**Supplementary Figures**

Volume rendering of the connected particles within the ROI scans of samples at the initial (a,c,e) and intermediate processing stages (b,d,f) imaged at Diamond Manchester Imaging Branchline I13-2 to supplement Figures 2 and 3. The scale bar corresponds to 20 µm.

**
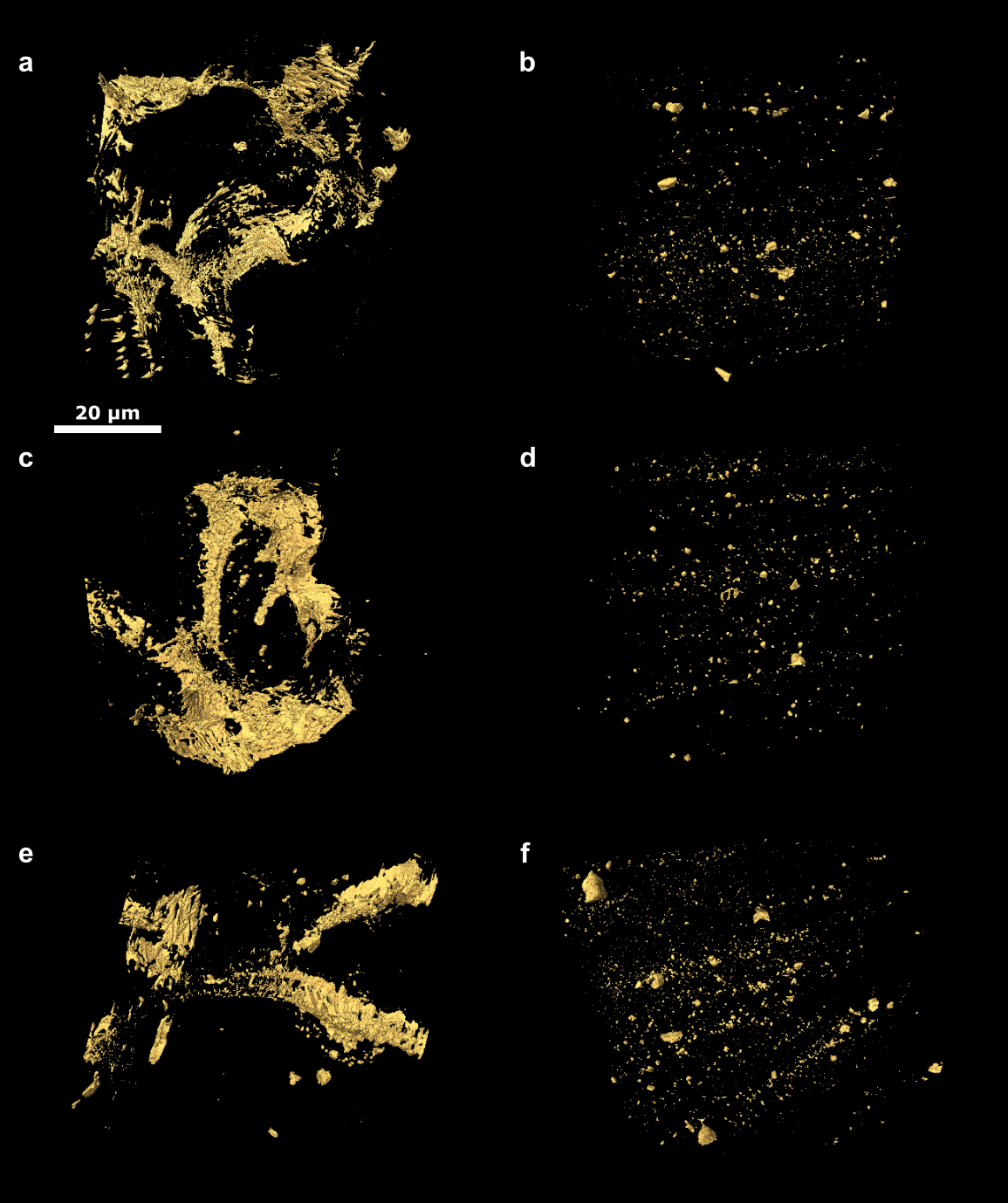
**
